# ddRAD sequencing based genotyping of six indigenous dairy cattle breeds of India to infer existing genetic diversity and population structure

**DOI:** 10.1038/s41598-023-32418-6

**Published:** 2023-06-09

**Authors:** Nampher Masharing, Monika Sodhi, Divya Chanda, Inderpal Singh, Prince Vivek, Manish Tiwari, Parvesh Kumari, Manishi Mukesh

**Affiliations:** 1 Animal Biotechnology Division, ICAR-National Bureau of Animal Genetic Resources, Karnal, Haryana India; 2 Animal Biotechnology Center, ICAR-National Dairy Research Institute, Karnal, Haryana India; 3 ICAR-NBAGR, Karnal, Haryana 132001 India

**Keywords:** Biotechnology, Genomics, Phylogenomics

## Abstract

The present investigation aimed to identify genome wide SNPs and to carry out diversity and population structure study using ddRAD-seq based genotyping of 58 individuals of six indigenous milch cattle breeds (*Bos indicus*) such as Sahiwal, Gir, Rathi, Tharparkar, Red Sindhi and Kankrej of India. A high percentage of reads (94.53%) were mapped to the *Bos taurus* (ARS-UCD1.2) reference genome assembly. Following filtration criteria, a total of 84,027 high quality SNPs were identified across the genome of 6 cattle breeds with the highest number of SNPs observed in Gir (34,743), followed by Red Sindhi (13,092), Kankrej (12,812), Sahiwal (8956), Tharparkar (7356) and Rathi (7068). Most of these SNPs were distributed in the intronic regions (53.87%) followed by intergenic regions (34.94%) while only 1.23% were located in the exonic regions. Together with analysis of nucleotide diversity (π = 0.373), Tajima’s D (D value ranging from − 0.295 to 0.214), observed heterozygosity (H_O_ ranging from 0.464 to 0.551), inbreeding coefficient (F_IS_ ranging from − 0.253 to 0.0513) suggested for the presence of sufficient within breed diversity in the 6 major milch breeds of India. The phylogenetic based structuring, principal component and admixture analysis revealed genetic distinctness as well as purity of almost all of the 6 cattle breeds. Overall, our strategy has successfully identified thousands of high-quality genome wide SNPs that will further enrich the *Bos indicus* representation basic information about genetic diversity and structure of 6 major Indian milch cattle breeds which should have implications for better management and conservation of valuable indicine cattle diversity.

## Introduction

The Indian subcontinent is home to a mega diverse *Bos indicus* cattle breeds of the world^1^. The Indian zebu cattle (*Bos Indicus*) is believed to have originated from wild aurochs *Bos primigenius nomadicus*^2,3^ and studies based on mitochondrial DNA markers analysis indicated that *Bos indicus* separated from *Bos taurus* between 110,000 and 850,000 years ago^4,5^. Worldwide, I1 and I2 are the two major mitochondrial DNA (mtDNA) haplogroups have been reported for *Bos indicus*. The I1 which is predominant haplogroup is believed to have originated from India–Pakistan, whereas haplogroup I2 has a complex diversity pattern making it difficult to resolve its origin^6–8^. Although recent findings have identified a new sub-haplogroup I1a, in the I1 haplogroup within the *Bos indicus* lineage^9^. On the other hand, the Y chromosome diversity found in *Bos indicus* cattle is characterized by a single haplogroup Y3, in contrast to two different haplogroups Y1 and Y2 found in *Bos taurus*. In addition, two distinct sub-haplogroups within each of the Y2 (Y2a and Y2b) and Y3 (Y3a and Y3b) haplogroups have been identified. The Y3 haplogroup was observed to be inimitable to *Bos indicus* and findings have shown that the sub-haplogroup Y3a dominated the cattle from South China, whereas the sub-haplotype Y3b was found in the *Bos indicus* breeds of Indian origin^10^. With a population of 192.49 million cattle, India has 13.1% of world’s cattle population^11^. Further, India holds the first rank in milk production in the world with a total production of 198.4 million tons of milk production during 2019–2020^12^. The Indian zebu cattle is an important member of the Bovidae family and is a major resource for milk and drought power in the Indian subcontinent. At present there are 53 well defined Indigenous cattle breeds in India which can be differentiated as dairy, dual or draft purpose breeds on the basis of their utility. The dairy cattle breeds on the average produced more than 1600 kg of milk per lactation, the dual-purpose breeds yield about 150–500 kg per lactation while the draft breeds are mainly used for agricultural work. The major dairy breeds of India include Gir (GIC), Rathi (RAC), Red Sindhi (RSC), Sahiwal (SAC) and Tharparkar (THC), the dual-purpose breeds comprise of Badri, Belahi, Deoni, Gaolao, Hariana, Kankrej, Konkani, Ladakhi, Malnad Gidda, Mewati, Ongole while the remaining breeds are classified as draft breeds.

The Indian native cattle breeds (*Bos indicus*) are well adapted to withstand the harsh climate and still perform efficiently. Besides, these are suited to low input production system with lower maintenance and management requirements. However, under the present production system, which is mostly focusing on increased milk production, the population size of indigenous cattle is in declining phase due to (1) modernization of agriculture and (2) cross breeding with exotic breeds to maximize the overall production and economic profit. The negligence of superior characters of Indian cattle like adaption to diverse climate and survival on low input system over production is resulting in loss of breeds or overall genetic diversity. Hence mitigation measures to characterize and conserve genetic diversity is key to evade further loss of important gene/gene pool and loss in the variability, which is very important for achieving higher genetic gain in economic traits of the indigenous dairy cattle breeds. In depth characterization and evaluation of genetic diversity among cattle breeds is of great importance to ensure long-term genetic improvement, facilitate rapid adaptation to changing climate and for efficient management and conservation of animal genetic resources^13,14^.

Genome-wide studies focused on population genetics, phylogeography and conservation biology have been greatly facilitated by quick advances in high-throughput sequencing technologies^15^. In recent years, reduced representation sequencing method such as double-digest restriction site associated DNA (ddRAD) approaches has received worldwide attention due to their capacity to identify genome-wide variations at relatively low cost. Genome wide SNPs based diversity and population structure analysis using ddRAD have been carried out in different livestock species like buffalo, yak, horse and camel^16–19^. ddRAD being a restriction digestion based reduced representation Next Generation Sequencing method fragments a target genome with both frequent and rare cutting restriction enzymes and such a strategy minimizes the hassles of uninformative and repetitive sequences, sequence assembly and SNP calling that accompanies with Whole genome sequencing (WGS). Evidently, ddRAD has been employed for the discovery of species-specific genome wide SNPs in economic, production and adaptation traits related candidate genes^20–22^. Furthermore, reduced representation methods based on whole genome sequencing of single individuals solve the problem of ascertainment bias^23^. Previously, array-based SNP chips has been widely used in genetic studies of livestock, including genome-wide association studies (GWAS)^24,25^, selection signature studies^26,27^, diversity and population structure analysis^28–30^. However, SNP chips commonly include SNPs that were previously discovered by DNA sequencing. These SNPs may not be geographically representative and tend to be at higher frequency than random SNPs and most importantly impair identification of casual mutations. Hence, population genetic parameters such as diversity, population structure and recombination estimates may be biased^23,31^. Therefore, in this study we have applied ddRAD sequencing approach, to overcome ascertainment biasness for discovering genome wide SNPs and undertake diversity studies in indicine cattle.

Considering the valuable contribution of the native dairy cattle in supporting the livelihood of many Indians for many generations, few efforts have been made to evaluate the genetic diversity and relationship in Indian cattle using genome wide SNPs. Comprehensive characterization on within-and between-breed genetic diversity of the Indian native cattle breeds to facilitate an effective and rational management is lacking. Exploration of the genetic diversity including population structure and admixture can expedite appropriate conservation programs. Deep and thorough understanding of indigenous genes/gene pool will help to understand the mechanism underlying important functional traits and help to meet the future production demands of the local people. The present investigation was undertaken to identify genome wide SNPs and assess the within and between breed genomic diversity and establish breed relationships and to assess their population structure.

## Results

### Quality control, alignment and SNP calling

**Figure 1:**
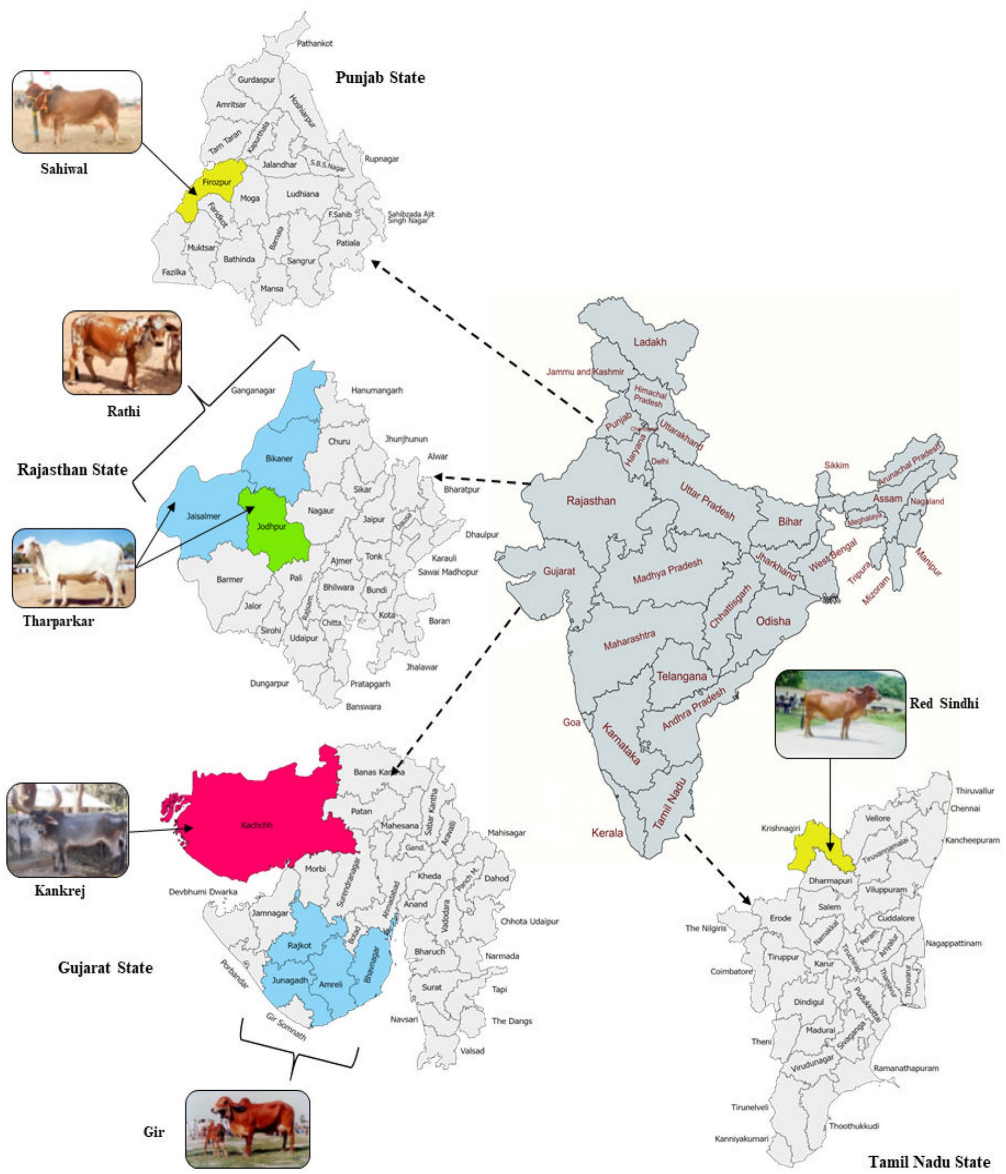
Geographical distribution of six cattle breeds included in this study (The map was generated using websites Map Chart https://www.mapchart.net/ and Paint Maps https://paintmaps.com/).

The ddRAD sequencing based genotyping of 58 individuals belonging to six native cattle breeds; Gir, Sahiwal, Tharparkar, Rathi, Red Sindhi and Kankrej cattle with their geographical and ecological distribution (Fig. 1) including the productive purpose, coat colour, representative agroclimatic zone, breeding tract, the geographical co-ordinate of each breeding tract along with animal ID and sex of each individual presented in Supplementary Table S1; resulted in 138.59 million raw reads that corresponded to 23 million reads per breed and 2.2 million reads per animal. After initial filtering on the basis of read quality and adaptor removal, majority of the reads (138.58 million reads; 99.9%) were retained (Supplementary Table S2). A high percentage of reads (94.53%) were mapped to the *Bos taurus* (ARS-UCD1.2) reference assembly (Supplementary Table S2). In this study, the effort was made to analyze only the SNPs across different cattle breeds, therefore all other variants were not considered in subsequent analysis. The number of SNPs in 6 cattle breeds ranged between 8,42,768 and 3,81,966 after individual variant calling. Maximum number of SNPs were observed in SAC (8,42,768), followed by GIC (8,34,780), KAC (8,10,279), RAC (8,05,020), RSC (6,72,632) and THC (3,81,966) (Table 1). The combined data set across 6 cattle breeds produced a total of 43,47,445 SNPs. Subsequently, the VCF file was processed in a stepwise manner to filter out low quality SNPs. Firstly, the SNPs were filtered at read depth of 2 (RD 2), read depth of 5 (RD 5) and read depth of 10 (RD 10). For further analysis, the data set of 9,82,174 SNPs identified at RD of 5, were utilized for subsequent analysis (Table 1). All those SNPs that were present at low coverage (RD < 5) were removed from the data set. The SNPs that were identified at RD of 5 were further filtered using various criteria’s such as proportion of missing genotypes, minor allele frequency and Hardy Weinberg Equilibrium (HWE). The series of filtering resulted in a total of 84,027 high quality SNPs. Post filtering, the number of SNPs across breeds varied considerably. The highest number of SNPs was observed in GIC (34,743), followed by RSC (13,092), KAC (12,812), SAC (8956), THC (7356) and RAC (7068) (Table 2).

**Table 1:** Number of SNPs identified at read depth (RD) of 2, 5 and 10 in 6 Indian cattle breeds.

Breeds	Raw SNPs	Only autosomes	RD 2	RD 5	RD 10
GIC	834,780	809,903	372,122	228,548	79,539
KAC	810,279	786,915	371,371	214,889	50,687
RAC	805,020	780,763	283,706	147,305	30,692
RSC	672,632	652,357	262,982	135,982	32,616
SAC	842,768	816,701	380,696	197,197	35,801
THC	381,966	372,730	168,195	58,253	9141
Total	43,47,445	42,19,369	18,39,072	9,82,174	2,38,476

*GIC Gir, *KAC* Kankrej, *RAC* Rathi, *RSC* Red Sindhi, *SAC* Sahiwal, *THC* Tharparkar.

**Table 2:** The number of high-quality SNPs in each cattle breed post series of filtering criteria.

	Filtering criteria			
Breeds	Genotype quality (GQ) ≥ 30	MAF ≤ 0.05	Missing genotype ≥ 80% HWE (*p* ≥ 0.001	LD pruned (r^2^ = 0.5)
GIC	2,28,548	1,91,761	34,743	22,594
KAC	2,14,889	1,79,350	12,812	8098
RAC	1,47,305	1,24,631	7068	4467
RSC	1,35,982	1,12,858	13,092	12,118
SAC	1,97,197	1,75,134	8956	5248
THC	58,253	16,549	7356	7252

*GIC Gir, *KAC* Kankrej, *RAC* Rathi, *RSC* Red Sindhi, *SAC* Sahiwal, *THC* Tharparkar.

### Functional annotation of variants

**Figure 2:**
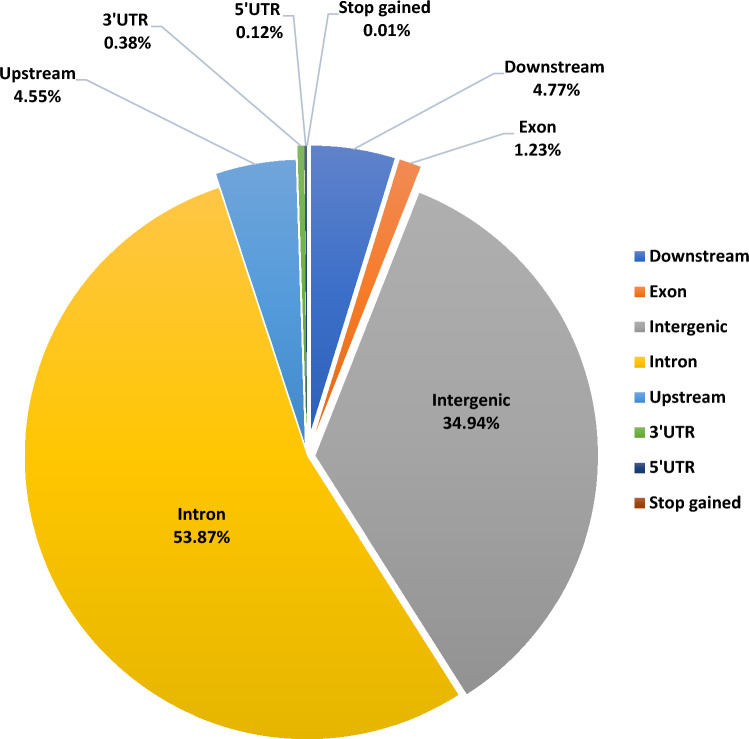
Overall partitioning of SNPs with respect to genomic distribution for all the breeds.

The merged high-quality SNPs dataset of all the 6 milch breeds was annotated to *Bos taurus* (*ARS-UCD1.2)* reference genome. With respect to their distribution in the genome, a large number of annotated SNPs were predicted to be in the intronic region (41,372 SNPs, 53.87%) followed by intergenic regions (26,834 SNPs, 34.94%). There were only 948 SNPs (1.23%) that were distributed in the exonic regions. Further, there were 3497 SNPs (4.55%) located within the 5 Kb region upstream and 3661 SNPs (4.77%) in the downstream of transcription start site. The analysis also resulted in 93 SNPs (0.121%) located in 5’UTR, 293 SNPs (0.38%) in 3’UTR region. A total of 8 SNPs (0.01%) were predicted to cause premature stop codon were also identified (Fig. 2).

**Figure 3:**
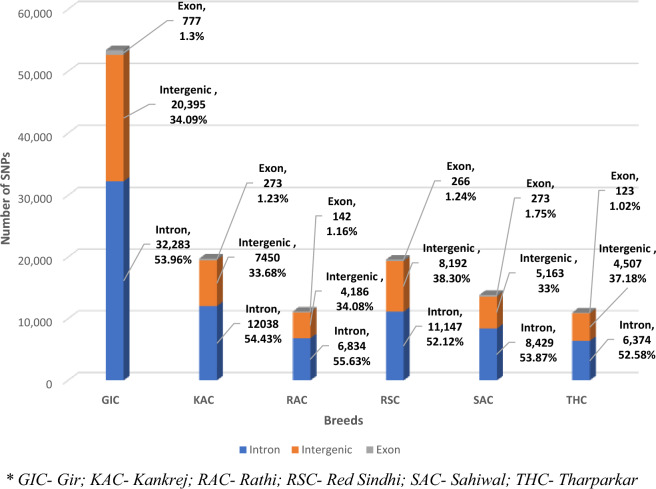
Genomic distribution of SNPs across the genome of six Indian milch cattle breeds.

On the basis of the impact of SNPs on protein coding genes, the SNPs were categorized as having high impact (10 SNPs; 0.01%), moderate impact (298 SNPs; 0.39%), and low impact, (697 SNPs; 0.91%). Majority of the SNPs (75,801; 98.69%) were identified as modifier (Supplementary Table S3). Additionally, high proportion of SNPs (65.74%) were silent in nature, followed by missense (33.37%) and nonsense (0.89%), with an average missense/silent ratio of 0.507 (Supplementary Table S4). In addition, amongst all genotypes substituted identified in the present study, C/T and G/A genotypes were found to be predominant, whereas A/T genotype was found to be in lowest proportions (Supplementary Table S5). For individual breed, the annotation results are summarized in Fig. 3 and Supplementary Table S6. In GIC, highest number of SNPs 32,283 (53.96%) were predicted to be in the intronic region followed by intergenic region 20,395 (34.09%). Only 777 (1.3%) were detected in the exonic region. Similar to GIC, the highest number of SNPs were distributed in intronic region followed by intergenic and exonic region in all other cattle breeds. For example, in SAC, 53.87% of SNPs (8429) were predicted in the intronic region followed by intergenic region 33% (5163 SNPs) and only 1.75% (273 SNPs) in exonic region. A similar trend was observed for RAC, RSC, KAC and THC cattle breeds with 6834 (55.63%), 11,147 (52.12%), 8429 (53.87%), 6374 (52.58%) SNPs, respectively in the intronic region, 4186 (34.08%), 8192 (38.30%), 5163 (33%), 4507 (37.18%) SNPs respectively, in the intergenic region and only 142 (1.16%), 266 (1.24%), 273 (1.75%), 123 (1.02%) were predicted in the exonic regions (Fig. 3). The number of synonymous variants identified in GIC, KAC, RAC, RSC, SAC and THC were 570, 190, 101, 172, 213 and 87 respectively. On the other hand, the number of non-synonymous variants detected for the 6 cattle breeds were 165, 64, 31, 82, 53 and 30 respectively. The T_S_/T_V_ ratio observed in GIC, KAC, RAC RSC SAC and THC was 2.55, 2.64, 2.33, 2.43, 2.51 and 2.19 respectively (Supplementary Table S6).

The numbers of intergenic SNPs were 4,639,873 (68.1%) and 1,676,710 (24.6%) were intronic. There were 230,365 (3.4%) SNPs located within 5 kb upstream and 197,827 (2.9%) in downstream of a transcription start site; 12,428 SNPs were located in the 5′ UTR and 2613 in the 3′ UTR. A total of 4356 SNPs were located in splice sites of 2966 genes: 142 were in splice-donor sites, 142 were splice-acceptor sites and 4072 were within the region of the splice site. We identified 45,776 SNPs affecting the coding sequences of 11,538 genes. There were 221 SNPs predicted to cause premature stop codon and 17 to cause gain in coding sequence. The numbers of SNPs predicted to be non-synonymous were 20,828. The numbers of intergenic SNPs were 4,639,873 (68.1%) and 1,676,710 (24.6%) were intronic. There were 230,365 (3.4%) SNPs located within 5 kb upstream and 197,827 (2.9%) in downstream of a transcription start site; 12,428 SNPs were located in the 5′ UTR and 2613 in the 3′ UTR. A total of 4356 SNPs were located in splice sites of 2966 genes: 142 were in splice-donor sites, 142 were splice-acceptor sites and 4072 were within the region of the splice site. The numbers of intergenic SNPs were 4,639,873 (68.1%) and 1,676,710 (24.6%) were intronic. There were 230,365 (3.4%) SNPs located within 5 kb upstream and 197,827 (2.9%) in downstream of a transcription start site; 12,428 SNPs were located in the 5′ UTR and 2613 in the 3′ UTR. A total of 4356 SNPs were located in splice sites of 2966 genes: 142 were in splice-donor sites, 142 were splice-acceptor sites and 4072 were within the region of the splice site. We identified 45,776 SNPs affecting the coding sequences of 11,538 genes. There were 221 SNPs predicted to cause premature stop codon and 17 to cause gain in coding sequence. The numbers of SNPs predicted to be non-synonymous were 20,828. The numbers of intergenic SNPs were 4,639,873 (68.1%) and 1,676,710 (24.6%) were intronic. There were 230,365 (3.4%) SNPs located within 5 kb upstream and 197,827 (2.9%) in downstream of a transcription start site; 12,428 SNPs were located in the 5′ UTR and 2613 in the 3' UTR. A total of 4,356 SNPs were located in splice sites of 2966 genes: 142 were in splice-donor sites, 142 were splice-acceptor sites and 4072 were within the region of the splice site. We identified 45,776 SNPs affecting the coding sequences of 11,538 genes. There were 221 SNPs predicted to cause premature stop codon and 17 to cause gain in coding sequence. The numbers of SNPs predicted to be non-synonymous were 20,828.

### Within breed diversity

The nucleotide diversity (π) was highest in THC (π = 0.458) followed by RSC (π = 0.364), SAC (π = 0.363), GIC (π = 0.356), KAC (π = 0.348) and RAC (π = 0.347). The mean nucleotide diversity value was 0.373 (Table 3). The Tajima’s D values were negative for 4 cattle breeds viz., RSC, RAC, SAC and THC except for GIC and SAC where positive D values was observed. The highest negative Tajima’s D value was observed in THC (-1.194) followed by RSC (− 1.088), RAC (− 0.295) and KAC (− 0.279).

**Table 3:** Nucleotide diversity and Tajima’s D values in six Indian milch cattle breeds.

Breeds	pi (π)	Tajima’s D
GIC	0.356	0.214
KAC	0.348	− 0.279
RAC	0.347	− 0.295
RSC	0.364	− 1.088
SAC	0.363	0.145
THC	0.458	− 1.19
Average	0.373	

*GIC Gir, *KAC* Kankrej, *RAC* Rathi, *RSC* Red Sindhi, *SAC* Sahiwal, *THC* Tharparkar.

The observed heterozygosity (H_O_) values ranged from 0.464 to 0.551 while the expected heterozygosity (H_E_) ranged from 0.448 to 0.535. The highest observed heterozygosity values were observed in THC (H_O_ = 0.551) followed by RAC (H_O_ = 0.523), RSC (H_O_ = 0.5184), SAC (H_O_ = 0.5180), GIC (H_O_ = 0.499) and KAC (H_O_ = 0.464) (Table 4). The average F_IS_ (inbreeding coefficient) ranges from -0.253 in THC to 0.0513 in KAC. The F_IS_ estimate amongst the six cattle breeds was highest in THC (F_IS_ = − 0.253) followed by RAC (F_IS_ = − 0.105), whereas the lowest F_IS_ estimate was observed in KAC (F_IS_ = 0.0513) followed by GIC (F_IS_ = − 0.00063). The overall F_IS_ analysis revealed excess of heterozygosity for all the cattle breeds except for KAC (Table 4). The heterozygosity and F_IS_ estimates indicated presence of sufficient diversity within the six cattle breeds.

**Table 4:** Within breed diversity statistics in six Indian milch cattle breeds.

Breeds	Obs het (H_O_)	Ex het (H_E_)	F_IS_
GIC	0.499	0.500	− 0.00063
KAC	0.464	0.535	0.0513
RAC	0.523	0.476	− 0.105
RSC	0.5184	0.481	− 0.079
SAC	0.5180	0.481	− 0.087
THC	0.551	0.448	− 0.253

*Obs Het observed heterozygosity, *Exp Het* expected heterozygosity, *GIC* Gir, *KAC* Kankrej, *RAC* Rathi, *RSC* Red Sindhi, *SAC* Sahiwal, *THC* Tharparkar.

### Between breed diversity

**Figure 4:**
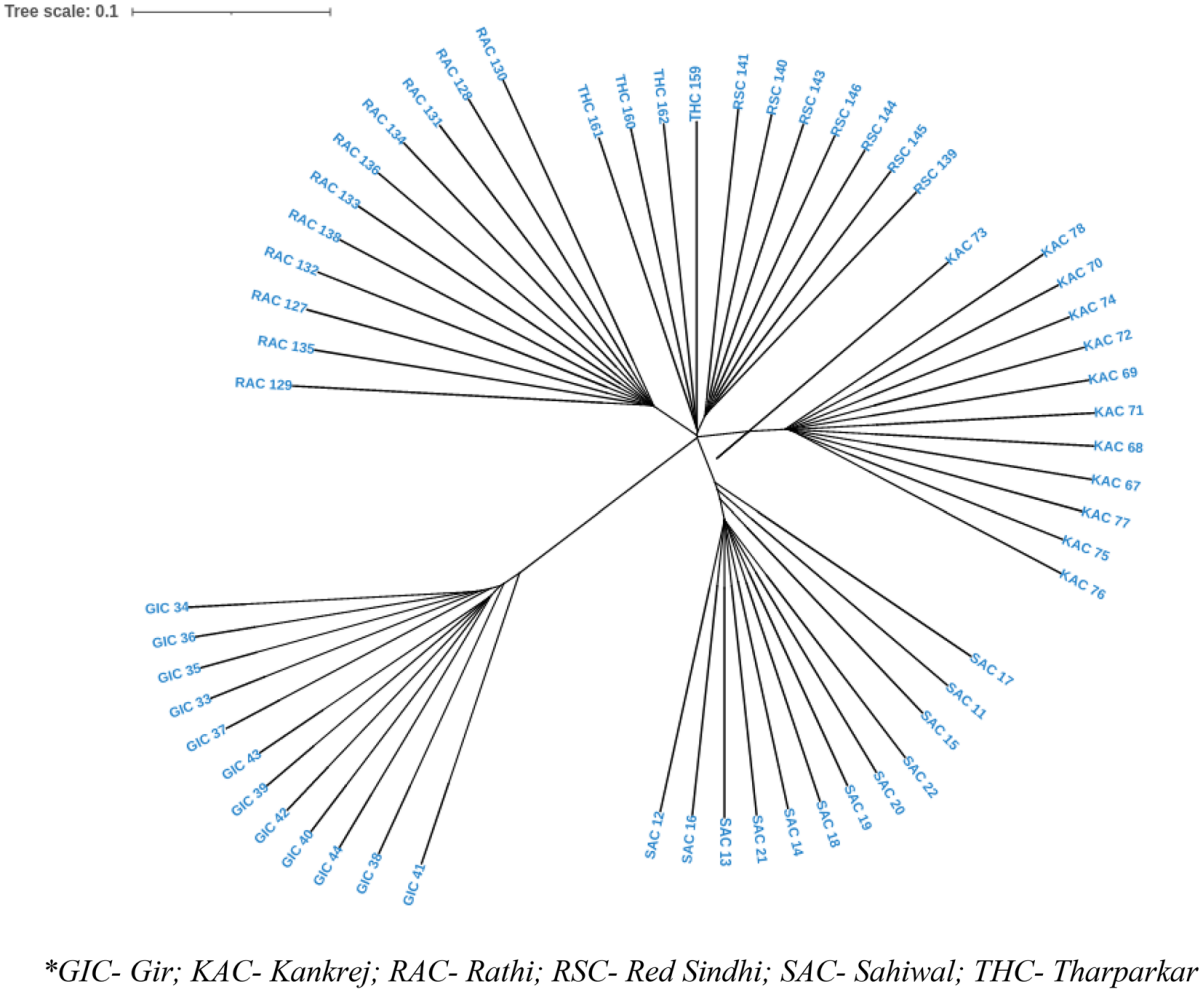
Neighbour-Joining based phylogenetic grouping of 58 animals of six Indian milch cattle breeds using Tassel software.

**Figure 5:**
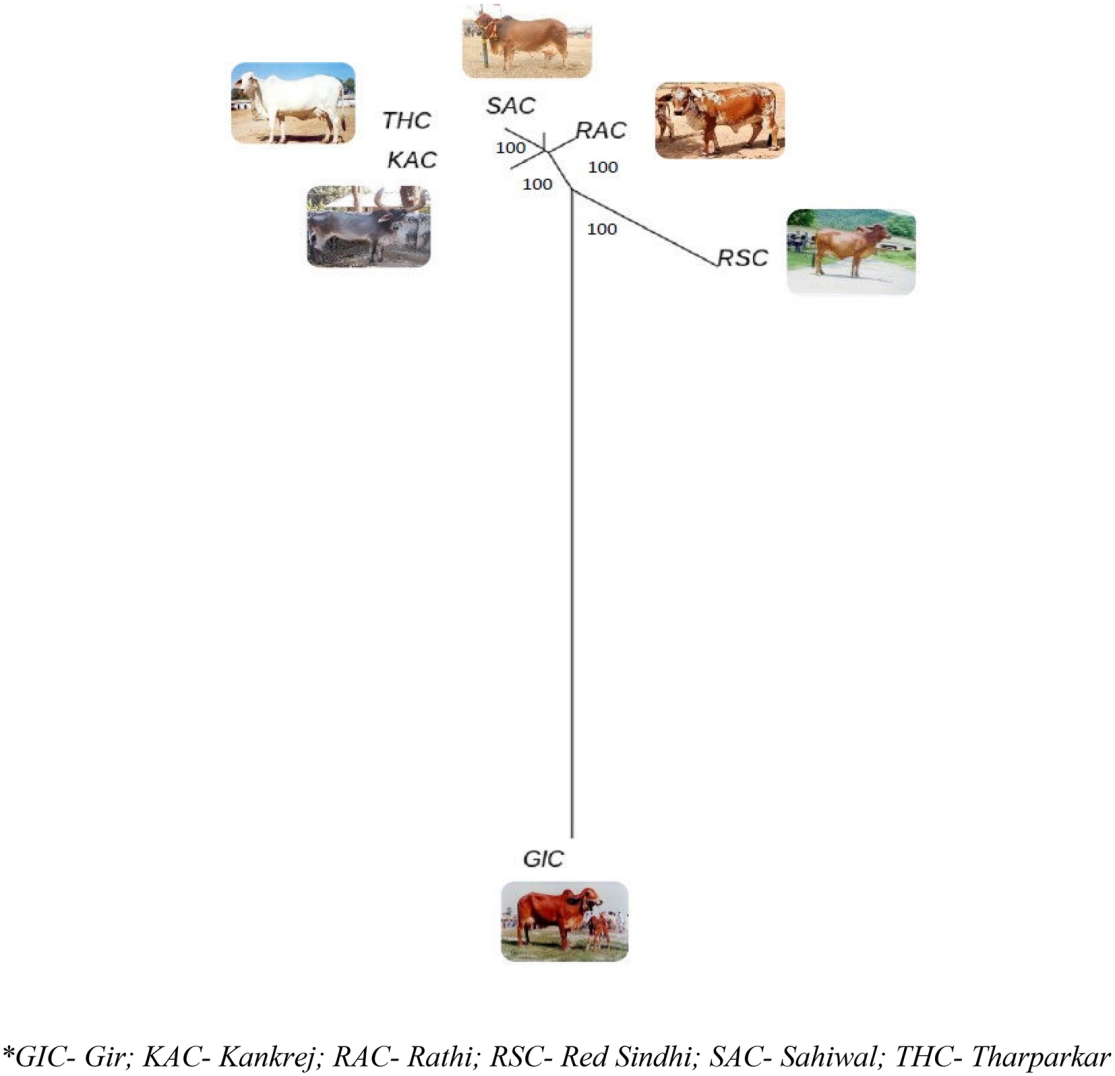
Neighbour-Joining based grouping of 6 Indian milch cattle breeds using “phangorn” package of R platform.

**Figure 6:**
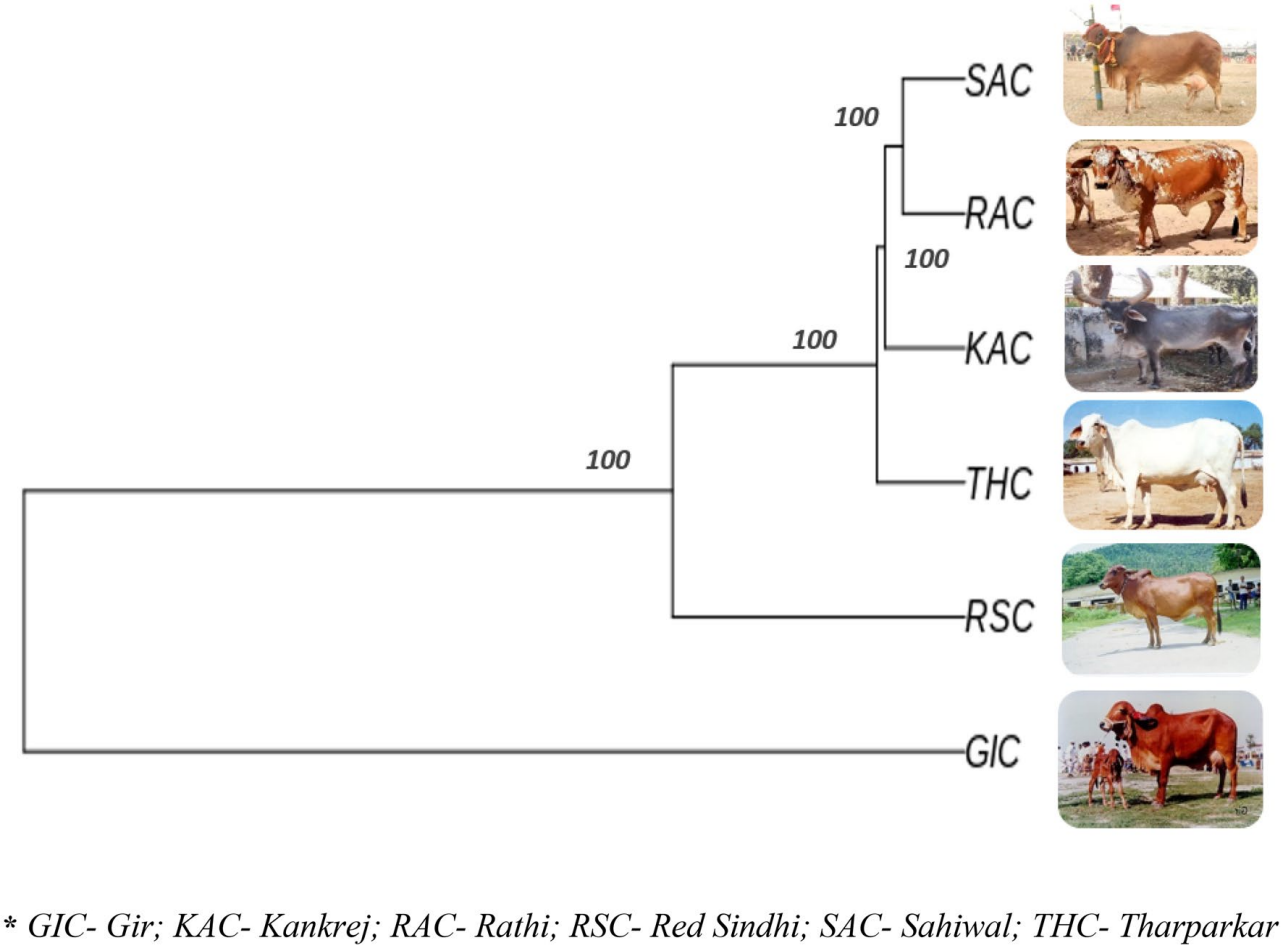
UPGMA based phylogenetic grouping of six Indian milch breed using “phangorn” package of R platform.

The genetic differentiation on the basis of fixation index (F_ST_) ranged from 0.2840 to 0.3905, indicating sufficient between breed diversity. The highest divergence was observed between RAC-SAC pair (F_ST_ = 0.3905), followed by RSC-RAC breed pair (F_ST_ = 0.3790), RSC-SAC breed pair (F_ST_ = 0.3751). The least divergence was observed for KAC-THC breed pair (F_ST_ = 0.2840) (Table 5). Neighbour Joining (NJ) based tree constructed, grouped the individual animals of 6 cattle breeds as per their breed affiliations with GIC and RSC being the most diverse breed amongst the 6 studied cattle breeds. The phylogenetic relationship at individual level is shown in Fig. 4. The breed wise NJ tree depicted in Fig. 5, more or less corroborated with the individual level tree. Furthermore, UPGMA based phylogenetic tree was constructed at breed level using “phangorn” package in R platform with 100 bootstrap values. The bootstrap values of each node were close to 100% indicating high robustness of the constructed tree. UPGMA based phylogenetic tree reflected similar genetic relationship as revealed by NJ based genetic differentiation (individual wise and at breed level) where GIC and RSC appeared as the most distinct breeds. GIC appeared on major node and clustered as one group while the other populations formed two groups with RSC clustered on one node and RAC, THC, SAC and KAC formed other sub clusters (Fig. 6).

**Table 5:** Pairwise F_ST_ statistics indicating genetic differentiation amongst the 6 Indian milch cattle breeds.

	SAC	GIC	THC	RAC	RSC	KAC
SAC	0	0.3705	0.3084	0.3905	0.3751	0.3654
GIC	0.3705	0	0.2876	0.3736	0.3402	0.3490
THC	0.3084	0.2876	0	0.3154	0.3496	0.2840
RAC	0.3905	0.3736	0.3154	0	0.3790	0.3672
RSC	0.3751	0.3402	0.3496	0.3790	0	0.3488
KAC	0.3654	0.3490	0.2840	0.3672	0.3488	0

*GIC Gir, *KAC* Kankrej, *RAC* Rathi, *RSC* Red Sindhi, *SAC* Sahiwal, *THC* Tharparkar.

### Population structure analysis

**Figure 7:**
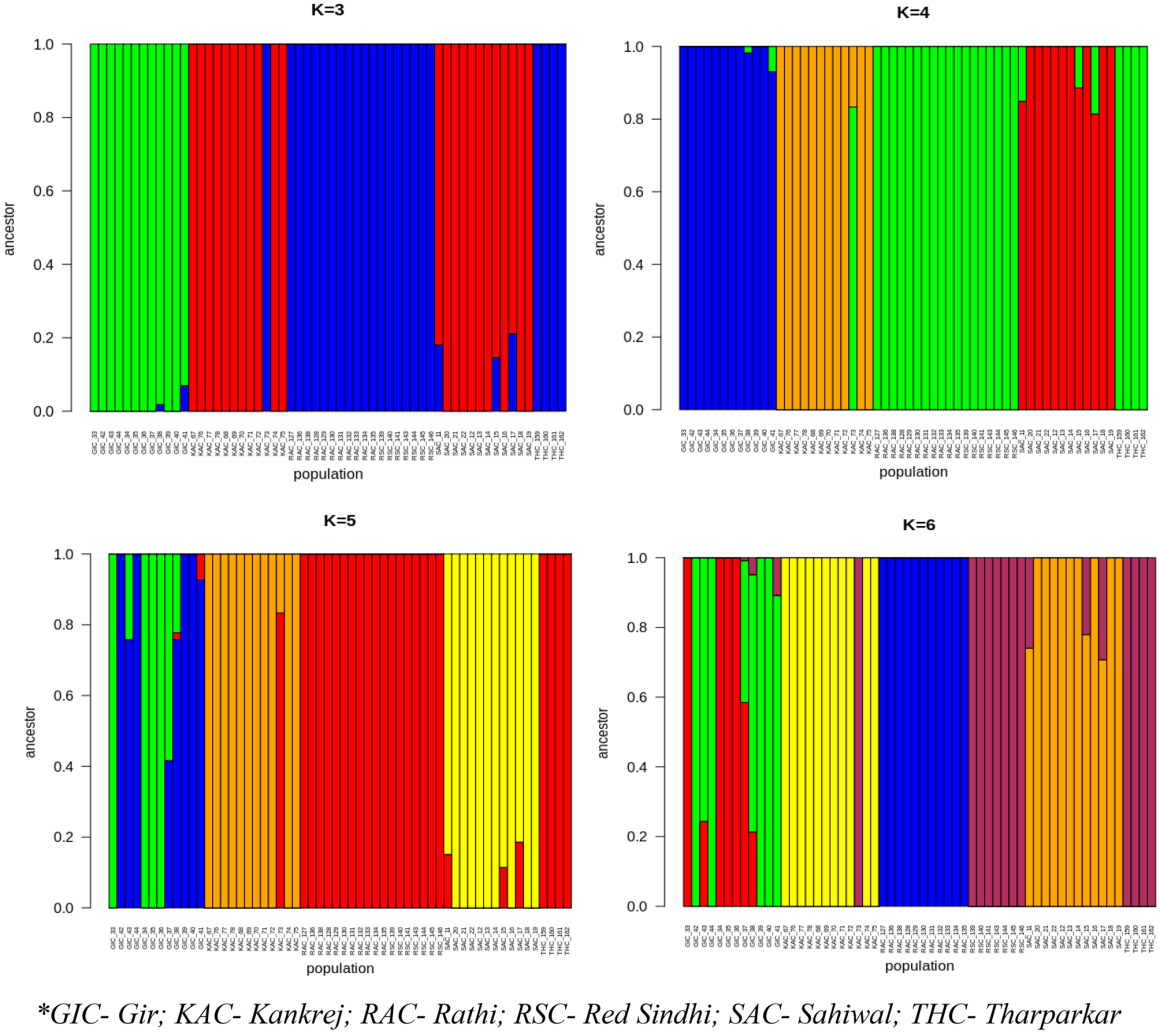
Admixture analysis assuming 3 ≤ K ≤ 6.

The admixture analysis was carried out by partitioning the genome of each individual into a predefined cluster. The analysis was performed at K = 3, 4, 5 and 6 (Fig. 7). The individuals could not be grouped at K = 3 as per their respective breed. Only GIC could distinctly be differentiated while the individuals of KAC and SAC appear as one group and RAC, THC and RSC are clustered together indicating their shared ancestry. At K = 4, and even at K = 5, THC, RAC and RSC clustered together indicating their strong shared ancestry, while all other individuals clustered in their own respective breed. The best K in population structure analysis is K = 6, whereby almost all the animals were grouped to their respective breed, clearly indicating their sperate ancestry, with the exception of RSC and THC which still clustered together. The genetic closeness between RSC and THC could be unveiled by further in-depth studies and by increasing the number of samples.

The PCA based analysis also clustered 6 cattle breeds separately and reinforces the fact that these are distinct cattle breeds (Supplementary Fig. S1). Individuals of KAC were grouped together in one quadrant, while individuals of SAC RAC, THC and RSC cattle breeds fall in a different quadrant. Individuals of GIC cattle breed appeared as a distinct population.

## Discussion

The Indian subcontinent is bestowed with immense richness of zebu (*Bos indicus*) cattle breeds. Geographical isolation over time has built up a plethora of genetic types/breeds but the magnitude of genetic differentiation has not been well quantified. Genetic variability in indigenous breeds is a major concern considering the necessity of preserving what may be a precious and irreplaceable richness developed as the results of complex interactions between the genotype and the environment. Hence molecular information is crucial for preserving genetic diversity as well as preventing undesirable loss of alleles. In this study genetic diversity and population structure of 6 major Indian milk cattle breeds was estimated using large number of genome wide SNPs generated through ddRAD sequencing.

In the present study, an average of 2.2 million reads per animal were obtained with a high mapping rate of 94.53% to *Bos taurus* reference genome (ARS-UCD1.2). The total number of SNPs identified in the present study varies when compared to previous reports. Gurgul et al.^32^ reported 8065 high-confidence SNPs in 48 individuals of different taurine cattle breeds by single enzyme restriction digestion GBS approach. Likewise, by using the same approach Malik et al.^33^ identified 1,07,488 SNPs in 24 animals belonging to seven Indian cattle breeds viz., Gangatiri, Hariana, Kankrej, Ongole, Sahiwal, Siri and Tharparkar. Furthermore, De Donato et al.^34^ identified 63,697 SNPs in 47 animals from both taurine and indicine breeds using a single enzyme GBS method. On the other hand, by using 2 enzyme GBS protocol, Brouard et al.^35^ reported a total of 2,72,103 variants in 48 Canadian dairy cattle. Similarly, RAD sequencing was used to identify 2,38,725 and 84,854 high-confidence SNPs in Sichuan and Liangshan indigenous cattle breeds of China, respectively^36,37^. Recently, studies carried out using ddRAD in Indian native cattle breed Sahiwal reported a total of 258,231 genome-wide SNPs with a minimum read depth of 2, 10, 232,570 SNPs at read depth 5 and 193,803 SNPs were identified respectively^21^. The number of high confidence SNPs identified in the present study and other previous studies could be attributed to the levels and stringency of filtering parameters applied while calling for SNPs.

The average Ts/Tv ratio was found to be 2.53. The observed Ts/Tv ratio in the present study was similar to many other reduced representation sequencing studies carried out in yak, buffaloes and cattle^17,38,39^. Large numbers of SNPs detected were found to be in the intronic and intergenic regions, which was similarly observed in previous studies^16,22^. Annotation of SNPs in this study also revealed that the G/A and C/T substituted genotypes were mostly found, whereas AT genotype was the least. This observation was similar to the studies carried out by Kumar et al.^11^ and Wang et al.^36^.

The nucleotide diversity (π) with an overall value of 0.373 was significantly higher in the 6 Indian native milch breeds when compared to the mean nucleotide diversity (π = 0.18 and π = 0.227) reported for Chinese cattle using RAD sequencing^36,37^. Furthermore, the nucleotide diversity in the studied cattle breeds was also comparatively high when compared to the nucleotide diversity reported for Eastern Finn cattle, Western Finn cattle, and Yakutian cattle with π values of 1.559 × 10^–3^, 1.512 × 10^–3^, and 1.728 × 10^–3^, respectively^40^. Similarly, other taurine breeds such as Braunvieh cattle of Switzerland also showed relatively lower nucleotide diversity^41^. The results of nucleotide diversity strongly suggest that the 6 major milch breed of India are maintaining sufficient degree of within breed genetic variation.

The negative Tajima’s D values observed for the 4 Indian cattle breeds viz., RSC, RAC, KAC and THC except for GIC and SAC signifies the population size expansion and presence of an excess of rare alleles. Further, the negative Tajima’s D values also signifies the occurrence of recent positive selection in these Indian native cattle breeds. This observation was also consistent with selection signals detected in some of the other SNP array-based studies involving Indian native breeds^26^ On the contrary the positive Tajima’s D values detected for Gir and Sahiwal indicates signals of balancing selection in these breeds. Similar observation was also reported whereby a lower selection signals in 7 Indian native breeds such as Gir, Hariana, Kankrej Ongole, Red Sindhi, Sahiwal and Tharpakar using 50 K bovine SNPchip data was identified^22^.

The observed (H_O_) and expected heterozygosity (H_E_) values for the 6 cattle breeds ranged from 0.464 to 0.551 (H_O_) and 0.448 to 0.535 (H_E_), respectively. The maximum heterozygosity value was observed in THC (H_O_ = 0.551) followed by RAC (H_O_ = 0.523), RSC (H_O_ = 0.5184), SAC (H_O_ = 0.5180), GIC (H_O_ = 0.499), while the lowest level of heterozygosity was observed in KAC (H_O_ = 0.464). The diversity estimates in the present study was much higher to what has been reported in seven taurine and indicine cattle breeds (0.064 to 0.197) from the US and Africa using GBS approach^34^. In addition, lower heterozygosity value (0.22) was also reported in Chinese cattle using restriction site-associated DNA sequencing (RADSeq)^37^. Further low diversity values were also reported by Malik et al.^33^ for two Indian *Bos indicus* cattle breeds; Sahiwal (H_O_ = 0.084) and Kankrej (H_O_ = 0.086) along with *Bos taurus* Holstein Frisian cattle^33^ using GBS approach. The explanation for lower heterozygosity in aforesaid mentioned studies could be due to use of single enzyme digestion in RADSeq and GBS approaches.

The overall F_IS_ analysis revealed significant deficit of inbreeding levels in all the cattle breeds under study (F_IS_ ranges from − 0.253 in THC to 0.0513) as high F_IS_ estimates is linked to high degree of inbreeding. The high negative F_IS_ values obtained in the study are similar to the study carried out by Strucken et al. (2021)^28^ in 13 Indian cattle breeds using 777 k SNP BovineHD Beadchip with exception for Sahiwal where indications of inbreeding are observed.

Sahiwal is an important and by far the best milch cattle breed of India, hence the race for rearing pure line animals with desired economic traits could have resulted in the slight increase in the F_IS_ estimate. However, the overall F_IS_ estimates observed are in contrast to those identified for Indian native cattle by microsatellite markers^42–44^ who reported high F_IS_ values. The depression in the F_IS_ estimate in the present investigation demonstrates the presence of heterozygote excess in the Indian native cattle breeds.

The fixation index (F_ST_) values that ranged from 0.2840 to 0.3905 suggested moderate to substantial genetic differentiation across the 6 cattle breeds. The maximum divergence was observed between RAC-SAC pair (FST = 0.3905), followed by RSC-RAC breed pair (FST = 0.3790), RSC-SAC breed pair (FST = 0.3751). The least divergence was observed for KAC-THC breed pair (FST = 0.2840). In few other studies based on bovine 50 K and 770 K SNP chips, the authors have reported relatively lower F_ST_ values for Indian cattle breeds^28,29^. The overall high between breed genetic differentiation in the present investigation might be attributed to the fact that the blood samples were obtained from individuals that are true to the breed and the studied populations are genetically distinct from each other.

The dendogram analysis on the basis of genetic distance and branch length revealed that all the 6 native cattle breeds clustered separately with GIC and RSC breed appeared to be the most distinct breeds. Our results are similar with the findings of Nayee et al.^29^ and Strucken et al.^28^, wherein they reported genetic distinctness of Gir and Red Sindhi from the other Indian native breeds. Furthermore, close groupings of RAC, THC, SAC and KAC were observed in the present study whereby, RAC and THC appeared to share a common evolutionary history.

The admixture analysis showed that all most all the 6 native cattle breeds have maintained their genetic purity with little traces of admixturing. At subpopulations K = 6, though few individuals of KAC and SAC were observed to have little admixturing from THC. The shared and common geographical area of these breeds and lack of pedigree information in the field conditions might have contributed to small extent of admixturing. Interestingly, at K = 6, a shared ancestry was observed between THC and RSC. Although in previous studies, no admixture analysis was carried out in both Tharparkar and Red Sindhi, our observation of genetic closeness between these breeds can be unraveled by further analysis as close geographical origins exists between the two breeds. Overall the admixture analysis in the present study was in agreement with one of our previous microsatellites based genotyping studies where in high proportions of individuals of native cattle have been assigned to their respective breeds^45^. Recently, Nayee et al.^29^ using 50 K and 770 K bovine HD SNP chips have also shown assignment of majority of the animals of Sahiwal, Gir, Kankrej to their respective breeds with minimal mixed ancestry. The genetic purity of Gir was also in agreement with one of the recent studies carried out by Strucken et al.^28^ using bovine HD 770 K SNP chip. Similarly, Dixit et al.^26^ have shown that genotyping with Illumina 50 K SNP chip resulted in clustering of most of animals (> 76%) of Gir, Sahiwal, Hariana, Ongole, Kangyam, into their respective breeds.

The genetic separation of these 6 native cattle breeds was also supported by principal component analysis. Except one individual of KAC, all the animals of GIC, KAC and SAC were grouped as per their breed affiliations and widely separated from each other. Similarly, the individuals of THC, RAC and RSC were also grouped as per their breed but were placed closed to each other. The phylogenetic, admixture and PCA analysis thus suggested substantial between breed genetic distinctness of the 6 major milch breeds of India. The outcome of the present study has lot of similarity with many other previous reports on Indian native cattle published either using microsatellite or SNP chip markers^26,28,29,43,45^.

The present study has shown the utility of ddRAD sequencing strategy in identifying thousands of high-quality SNPs in native milch cattle breeds of India. The study has also provided an opportunity to establish a robust methodology as well as bioinformatics pipeline to generate and characterize genome wide SNPs. The SNPs derived from genome of native cattle could also help to enrich the *Bos indicus* genome database for future exploitation in diversity and genotype: phenotype association studies. Further, the genome wide SNP data set has provided a strong clue that each of the 6 major milch cattle breeds of India has sufficient within breed diversity. The between breed analysis along with phylogenetic, admixture and PCA analysis showed high level of genetic distinctness and purity of each of the 6 cattle breeds. In future similar approach could be extended to rest of the native cattle breeds (*Bos indicus*) to define the population structure along with their evolutionary relationships. Further, as the native Indian cattle breeds are known for better milk quality, heat tolerance and disease resistance, therefore such data set could also be exploited to understand the signatures of selection with respect to these traits. Such information will be quite helpful to realize the potential of these tropically adapted native germplasm especially in the era of climate change and global warming.

## Methods

### Sample source and DNA extraction

To identify the genome wide SNPs, the blood samples of 58 unrelated animals belonging to Gir (GIC, n = 12), Sahiwal (SAC, n = 12), Kankrej (KAC, n = 12), Rathi (RAC, n = 11), Red Sindhi (RSC, n = 7), and Tharparkar (THC, n = 4) cattle breeds were collected by visiting their respective breeding tracts. However, the samples of Red Sindhi animals were collected from Hosur farm of Krishnagiri district of Tamil Nadu state as this breed is only available in organized cattle farms. The blood samples were collected as per the guidelines of Institutional Animal Ethics Committee (IAEC). Further, all the details related to animals’ experiments were as per the ARRIVE guidelines and all the procedures were approved by the animal ethics committee of ICAR-NBAGR, Karnal. The geographical and ecological distribution of the cattle breeds is shown in Fig. 1. The utility type, coat colour, representative agroclimatic zone, breeding tract and geographical co-ordinates of each breeding tract is presented in Supplementary Table S1. Fresh blood samples (8–9 ml) collected in EDTA vacutainer tubes by jugular vein puncture were stored at − 20 °C until genomic DNA extraction. Genomic DNA was isolated from whole blood using phenol–chloroform extraction method^46^ followed by purification through RNAse treatment and Qiaquick Nuclease Removal Kit (Qiagen, Valencia, CA) to eliminate any RNA related impurities. The quality of DNA was checked on agarose gel (1%) electrophoresis, and the quantity of DNA was measured using a Nanodrop Spectrophotometer (Nanodrop ND-1000).

### ddRAD library preparation and sequencing

For DNA library preparation, each sample was digested with two Restriction enzymes (REs); a 6 cutter EcoR1 (G/AATTC) and a 4 cutter Mse1 (T/TAA) (New England Biolabs, Ipswich,MA, USA) as determined by *in-silico* simulation using SimRAD package^47^. Briefly, 0.3–0.6 μg of genomic DNA of each animal was digested with the optimized restriction enzyme set. After digestion each end of digested fragment was ligated to EcoRI-specific P1 and the MseI-specific P2, barcoded adapters with a T4 DNA Ligase (New England Biolabs, Ipswich, MA, USA). The ligation reaction consisted of overnight incubation (> 12 h) at room temperature (approx. 21 °C) and heat deactivation of the enzyme at 65 °C for 10 min. In order to eliminate unincorporated adapters and small DNA fragments, ligation reactions were purified using with 0.8X volume of Agencourt AMPure XP SPRI magnetic beads (Beckman Coulter Life Sciences, Indianapolis, USA). A unique combination of the dual-indexed barcodes was attached to purified fragments with 14 cycles of PCR. Indexed PCR products were pooled in equal volumes and size selected using Agencourt AMPure XP SPRI magnetic beads. The amplification protocol involved initial denaturation at 95 °C for 3 min; 25 cycles of denaturation at 95 °C for 30 s, annealing at 55 °C for 30 s, and extension at 72 °C for 30 s; followed by a final extension at 72 °C for 5 min.

In the present study, a total of 2 sequencing libraries viz., *FGBS20H000717-1a, FGBS20H000718-1a* which included all the samples of different cattle breeds were constructed for sequencing on Illumina HiSeq™ 2000 sequencing platform. The concentration of each library was checked using Qubit^®^ 2.0 fluorometer (Thermo Fisher, Waltham, MA, USA). Each library was diluted to 1 ng/ul and the insert size was assessed and quantified using the Agilent high-sensitivity DNA kit in a 2100 Bioanalyzer (Agilent Technologies, CA, USA). Quantitative real-time PCR (qPCR) was performed to detect the effective concentration of each library. Finally, the libraries with appropriate insert size and effective concentration of more than 2 nM were sequenced on Illumina HiSeq™ 2000 and more than 100 bp end reads were generated.

### Quality checking, filtering of raw reads and SNPs identification

The raw paired-end FASTQ sequencing files were quality checked using FASTQC software^48^. The raw reads with less than Q20 were removed from the data set using PRINSEQ software^49^. Both the adaptors were removed using Cutadapt 1.15^50^. Finally, the filtered reads with read length of 144 bp were retained for subsequent analysis. The filtered reads were aligned to *Bos taurus* reference genome *ARS-UCD1.2* using Bowtie2 tool^51,52^. The aligned SAM files were converted to BAM files using SAMtools^53^ and subsequently sorted using Picard tool. All the duplicate reads were flagged and tagged using MarkDuplicatesWithMateCigar module of Picard tools^ 54^. The reads were re-calibrated using GATK-BQSR tool with default parameters^54^. In order to discover genome wide SNPs in each animal, GATK Haplotypecaller was run in ERC GVCF mode^54^. The breed wise cohort GVCF file was created by combining all the individual GVCF files using CombinedGVFs of GATK tool. The cohort GVCF files were converted to VCF using GenotypeGVCF command of GATK tool. The insertions and deletions (INDELS) were discarded using GATKSelect Variants. The SNPs were annotated with reference to 1000 Bull Genome data using BCFTools^ 55^. After annotation, all those SNPs located on X, Y chromosomes as well as mitochondrial DNA were removed using VCFtools^56^ and only those SNPs which are present in autosomes were retained for further analysis. The SNPs were also filtered at a minimum read depth level of 2, 5 and 10 (RD) and SNPs identified at RD of 5 were further filtered having minimum quality score of GQ30 using VCFtools^56^. Finally, three rounds of filtering for minor allele frequency (MAF < 0.05), missing genotypes (0.8), and HWE deviation (*P* < 0.001) was carried out using PLINK 1.9 ^57^ to retain the high-quality SNPs for downstream analysis.

### Annotation of SNP sites

The high-quality SNPs identified in each breed was annotated using SnpEff Ver. 4.3 software^58^. The VCF file and annotation data of the *Bos taurus* reference genome were used to partitioned the SNPs as per their genomic location such as exonic, intronic, upstream/downstream regions, splicing sites and intergenic regions. The SNPs were also categorized based on their functional impact on protein coding genes such as high, moderate, modifier, missense, nonsense and silent.

### Diversity and Population structure analysis

Nucleotide diversity (π), TajimaD in each of the 6 cattle breeds was computed using TASSEL software (v. 5.0)^59^ software by selecting 500-SNP sliding window with step size of 100-SNP. VCF tools was employed to calculate observed heterozygosity (H_O_), expected heterozygosity (H_E_), inbreeding co-efficient (F_IS_) and Wright's F_ST_ estimates. For phylogenetic relationship, amongst the studied cattle populations, TASSEL software (v. 5.0)^59^ and phangorn^60^ softwares available in R language were used. A bootstrap value of 100 was used to draw the tree based on UPGMA and Neighbor joining (NJ) algorithms. The SNPs that were in strong Linkage Disequilibrium (LD) (r^2^ > 0.5) in a 5000-Kb sliding windows with 50 SNPs were pruned using *PLINK* v.1.9^57^. The admixture analysis was performed using the pruned SNPs data by applying the ADMIXTOOLS of admixr-R package^61^. The admixture analysis was performed by assuming different numbers of sub-populations K = 6 in order to identify the optimal number of ancestral populations by detecting the lowest value of cross-validation error. Similarly, the Principal component analysis (PCA) was performed using pruned data by employing “adegent” software and the results were plotted using ggplot.

### Animal ethics

All the experimental procedure was done in accordance with the ARRIVE guidelines and regulations of Institutional Animal Ethics Committee (IAEC), ICAR-National Bureau of Animal Genetic Resources (ICAR-NBAGR), Karnal, Haryana, India.

## Supplementary Information

Supplementary Information.
