# Supplementary material for: ddRAD sequencing based genotyping of six indigenous dairy cattle breeds of India to infer existing genetic diversity and population structure
Source: Sci Rep. 2023 Jun 9;13:9379. doi: 10.1038/s41598-023-32418-6 (PMC10256769; doi:10.1038/s41598-023-32418-6)
Supplement: Supplementary file 1 — Supplementary Information. [file 41598_2023_32418_MOESM1_ESM.docx]

**Supplementary Table S1**

List of Indian cattle breeds along with their productive purpose, coat colour, agroclimatic zone, the geographical co-ordinate of each breeding tract included in the present study.

| **Breeds** | **Acronym** | **Productive Purpose** | **Coat Colour** | **GR^a^** | **AZ^b^** | **State** | **Breeding tracts** | **Geographic co-ordinates** | **Samples Number** | **Animal ID** | **Sex** |
| --- | --- | --- | --- | --- | --- | --- | --- | --- | --- | --- | --- |
| Gir | GIC | Dairy | Red | NWR | SAR | Gujarat | Amreli, Juganadh, Bhavnagar, Rajkot | 21.6015° N, 71.2204° E | 1 | GIC_33 | M |
|  |  |  |  |  |  |  |  |  | 2 | GIC_34 | F |
|  |  |  |  |  |  |  |  |  | 3 | GIC_35 | M |
|  |  |  |  |  |  |  |  |  | 4 | GIC_36 | F |
|  |  |  |  |  |  |  |  |  | 5 | GIC_37 | M |
|  |  |  |  |  |  |  |  |  | 6 | GIC_38 | F |
|  |  |  |  |  |  |  |  |  | 7 | GIC_39 | F |
|  |  |  |  |  |  |  |  |  | 8 | GIC_40 | F |
|  |  |  |  |  |  |  |  |  | 9 | GIC_41 | F |
|  |  |  |  |  |  |  |  |  | 10 | GIC_42 | F |
|  |  |  |  |  |  |  |  |  | 11 | GIC_43 | F |
|  |  |  |  |  |  |  |  |  | 12 | GIC_44 | M |
| Kankrej | KAC | Dual purpose | Grey/White | NWR | SAR | Gujarat | Rann of Kuchh area comprising of Mehsana, Kutch, Ahmedabad, Sabarakantha | 24.0454° N, 70.1456° E | 13 | KAC_67 | F |
|  |  |  |  |  |  |  |  |  | 14 | KAC_68 | F |
|  |  |  |  |  |  |  |  |  | 15 | KAC_69 | M |
|  |  |  |  |  |  |  |  |  | 16 | KAC_70 | F |
|  |  |  |  |  |  |  |  |  | 17 | KAC_71 | M |
|  |  |  |  |  |  |  |  |  | 18 | KAC_72 | F |
|  |  |  |  |  |  |  |  |  | 19 | KAC_73 | F |
|  |  |  |  |  |  |  |  |  | 20 | KAC_74 | M |
|  |  |  |  |  |  |  |  |  | 21 | KAC_75 | F |
|  |  |  |  |  |  |  |  |  | 22 | KAC_76 | M |
|  |  |  |  |  |  |  |  |  | 23 | KAC_77 | F |
|  |  |  |  |  |  |  |  |  | 24 | KAC_78 | F |
| Rathi | RAC | Dairy | Brown with white patches | NWR | AR | Rajasthan | Bikaner, Jaislamer, Ganganagar | 26.2389° N, 73.0243° E | 25 | RAC_127 | F |
|  |  |  |  |  |  |  |  |  | 26 | RAC_128 | F |
|  |  |  |  |  |  |  |  |  | 27 | RAC_129 | F |
|  |  |  |  |  |  |  |  |  | 28 | RAC_130 | F |
|  |  |  |  |  |  |  |  |  | 29 | RAC_131 | M |
|  |  |  |  |  |  |  |  |  | 30 | RAC_132 | M |
|  |  |  |  |  |  |  |  |  | 31 | RAC_133 | F |
|  |  |  |  |  |  |  |  |  | 32 | RAC_134 | F |
|  |  |  |  |  |  |  |  |  | 33 | RAC_135 | F |
|  |  |  |  |  |  |  |  |  | 34 | RAC_136 | M |
|  |  |  |  |  |  |  |  |  | 35 | RAC_138 | M |
| Red Sindhi | RSC | Dairy | Red | SR | TWDR | Tamil Nadu | Krishnagiri, Organised farm, located at Hosur | 12.7409° N, 77.8253° E | 36 | RSC_139 | F |
|  |  |  |  |  |  |  |  |  | 37 | RSC_140 | F |
|  |  |  |  |  |  |  |  |  | 38 | RSC_141 | F |
|  |  |  |  |  |  |  |  |  | 39 | RSC_143 | F |
|  |  |  |  |  |  |  |  |  | 40 | RSC_144 | F |
|  |  |  |  |  |  |  |  |  | 41 | RSC_145 | F |
|  |  |  |  |  |  |  |  |  | 42 | RSC_146 | F |
| Sahiwal | SAC | Dairy | Reddish dun | NR/NWR | AR/SAR | Punjab | Ferozpur | 30.9331° N, 74.6225° E | 43 | SAC_11 | F |
|  |  |  |  |  |  |  |  |  | 44 | SAC_12 | F |
|  |  |  |  |  |  |  |  |  | 45 | SAC_13 | M |
|  |  |  |  |  |  |  |  |  | 46 | SAC_14 | F |
|  |  |  |  |  |  |  |  |  | 47 | SAC_15 | F |
|  |  |  |  |  |  |  |  |  | 48 | SAC_16 | F |
|  |  |  |  |  |  |  |  |  | 49 | SAC_17 | M |
|  |  |  |  |  |  |  |  |  | 50 | SAC_18 | F |
|  |  |  |  |  |  |  |  |  | 51 | SAC_19 | M |
|  |  |  |  |  |  |  |  |  | 52 | SAC_20 | M |
|  |  |  |  |  |  |  |  |  | 53 | SAC_21 | F |
|  |  |  |  |  |  |  |  |  | 54 | SAC_22 | F |
| Tharparkar | THC | Dairy | Grey/White | NWR | SAR | Rajasthan | Jodhpur, Jaislamer | 26.2389° N, 73.0243° E | 55 | THC_159 | M |
|  |  |  |  |  |  |  |  |  | 56 | THC_160 | F |
|  |  |  |  |  |  |  |  |  | 57 | THC_161 | F |
|  |  |  |  |  |  |  |  |  | 58 | THC_162 | F |

***^a^Geographical regions: NR-northern region; NWR-north western region; SR-southern region. ^b^Agroclimatic zones: SAR-semi arid; AR-arid; TWDR-tropical wet and dry.***

**Supplementary Table S2**

Reads before and after Quality checking (QC) and Breed wise Mapping statistics

| Breeds | Number of samples | Raw reads | QC Processed reads | Mapped Reads (%) | Unmapped reads (%) | Coverage Mean | Mean Mapping Quality |
| --- | --- | --- | --- | --- | --- | --- | --- |
| GIC | 12 | 3,44,36,538 | 3,44,33,382 | 98.36% | 1.64% | 0.14902 | 7.18 |
| KAC | 12 | 3,00,89,678 | 3,00,87,926 | 97.57% | 2.43% | 0.12879 | 6.956 |
| RAC | 11 | 2,51,34,412 | 2,51,34,412 | 88.91% | 11.09% | 0.11 | 7.023 |
| SAC | 7 | 1,53,65,736 | 1,53,65,736 | 98.91% | 1.09% | 0.11132 | 6.715 |
| RSC | 12 | 2,71,52,526 | 2,71,51,244 | 97.10% | 2.90% | 0.11 | 6.734 |
| THC | 4 | 6413700 | 6413700 | 86.33% | 13.67% | 0.08 | 6.5 |
| Average | 58 | 13,85,92,590 | 13,85,86,400 | 94.53% | 5.47% | 0.11 | 6.85 |

** GIC- Gir; KAC- Kankrej; RAC- Rathi; RSC- Red Sindhi; SAC- Sahiwal; THC- Tharparkar*

**Supplementary Table S3**

SNPs impact on protein coding

|  | Count | Percentage |
| --- | --- | --- |
| High | 10 | 0.01 |
| Low | 697 | 0.91 |
| Moderate | 298 | 0.39 |
| Modifier | 75,801 | 98.69 |

**Supplementary Table S4**

Types of mutation

|  | Count | Percentage |
| --- | --- | --- |
| Missense | 299 | 33.37 |
| Nonsense | 8 | 0.89 |
| Silent | 589 | 65.74 |
| Missense/ Silent Ratio | 0.507 | |

**Supplementary Table S5**

Changes in nucleotide bases across six Indian dairy cattle breeds

|  | A | C | G | T |
| --- | --- | --- | --- | --- |
| A | 0 | 1,664 | 7,067 | 1,087 |
| C | 1,743 | 0 | 1,867 | 9,063 |
| G | 8,979 | 1,917 | 0 | 1,785 |
| T | 1,123 | 7,020 | 1,729 | 0 |

**Supplementary Table S6**

Genomic distribution and nature of annotated SNPs in each of the six Indian dairy cattle breeds

|  | Breeds | | | | | |
| --- | --- | --- | --- | --- | --- | --- |
| Types | GIC | KAC | RAC | RSC | SAC | THC |
| Intron | 32283 (53.96%) | 12038 (54.43%) | 6834 (55.63%) | 11147 (52.12%) | 8429 (53.87%) | 6374 (52.58%) |
| Intergenic | 20395 (34.09%) | 7450 (33.68%) | 4186 (34.08%) | 8192 (38.30%) | 5163 (33%) | 4507 (37.18%) |
| Exon | 777 (1.3%) | 273 (1.23%) | 142 (1.16%) | 266 (1.24%) | 273 (1.75%) | 123 (1.02%) |
| Non-synonymous variant | 165 | 64 | 31 | 82 | 53 | 30 |
| Synonymous variant | 570 | 190 | 101 | 172 | 213 | 87 |
| Transitions | 1,36,699 | 46,494 | 23,001 | 29,818 | 34,884 | 10,088 |
| Transversions | 53,561 | 17,605 | 9,866 | 12,242 | 13,882 | 4,586 |
| Ts/Tv ratio | 2.55 | 2.64 | 2.33 | 2.43 | 2.51 | 2.19 |

** GIC- Gir; KAC- Kankrej; RAC- Rathi; RSC- Red Sindhi; SAC- Sahiwal; THC- Tharparkar*


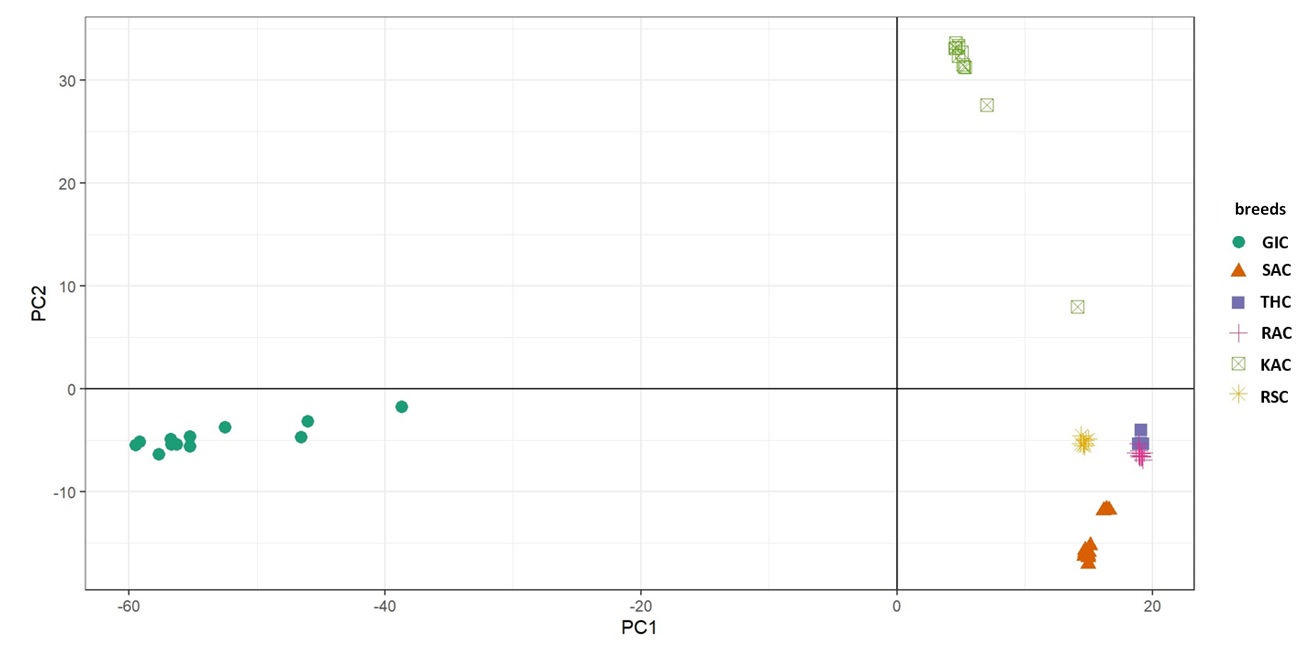


** GIC- Gir; KAC- Kankrej; RAC- Rathi; RSC- Red Sindhi; SAC- Sahiwal; THC- Tharparkar*

**Supplementary Fig. S1: Principal component analysis in six Indian dairy cattle breeds**
